# Local Electronic Structure in AlN Studied by Single-Crystal ^27^Al and ^14^N NMR and DFT Calculations

**DOI:** 10.3390/molecules25030469

**Published:** 2020-01-22

**Authors:** Otto E. O. Zeman, Igor L. Moudrakovski, Carsten Hartmann, Sylvio Indris, Thomas Bräuniger

**Affiliations:** 1 Department of Chemistry, University of Munich (LMU), Butenandtstr. 5-13, 81377 Munich, Germany; o.zeman@campus.lmu.de; 2 Max-Planck-Institut for Solid-State Research, Heisenbergstrasse 1, 70569 Stuttgart, Germany; I.Moudrakovski@fkf.mpg.de; 3 Leibniz-Institut für Kristallzüchtung (IKZ), Max-Born-Str. 2, 12489 Berlin, Germany; carsten.hartmann@ikz-berlin.de; 4 Karlsruhe Institute of Technology, Institute for Applied Materials (IAM), Hermann-von-Helmholtz-Platz 1, 76344 Eggenstein-Leopoldshafen, Germany

**Keywords:** AlN, single-crystal NMR, ^14^N NMR, ^27^Al NMR, chemical shift tensor, quadrupole coupling tensor

## Abstract

Both the chemical shift and quadrupole coupling tensors for ${}^{14}$N and ${}^{27}$Al in the wurtzite structure of aluminum nitride have been determined to high precision by single-crystal NMR spectroscopy. A homoepitaxially grown AlN single crystal with known morphology was used, which allowed for optical alignment of the crystal on the goniometer axis. From the analysis of the rotation patterns of ${}^{14}$N (I=1) and ${}^{27}$Al (I=5/2), the quadrupolar coupling constants were determined to $\chi {(}^{14}N)=\left(8.19\pm 0.02\right)$ kHz, and $\chi {(}^{27}\mathrm{Al})=\left(1.914\pm 0.001\right)$ MHz. The chemical shift parameters obtained from the data fit were $\delta_{iso}=-\left(292.6\pm 0.6\right)$ ppm and $\delta_{\Delta }=-\left(1.9\pm 1.1\right)$ ppm for ${}^{14}$N, and (after correcting for the second-order quadrupolar shift) $\delta_{iso}=\left(113.6\pm 0.3\right)$ ppm and $\delta_{\Delta }=\left(12.7\pm 0.6\right)$ ppm for ${}^{27}$Al. DFT calculations of the NMR parameters for non-optimized crystal geometries of AlN generally did not match the experimental values, whereas optimized geometries came close for ${}^{27}$Al with ${\bar{\chi }}_{\mathrm{calc}}=\left(1.791\pm 0.003\right)$ MHz, but not for ${}^{14}$N with ${\bar{\chi }}_{\mathrm{calc}}=-\left(19.5\pm 3.3\right)$ kHz.

## 1. Introduction

Aluminum nitride, AlN, is industrially used as a substrate for semiconductor devices such as ultraviolet LEDs, and is also the preferred starting material for the synthesis of chemically inert lightweight ceramics with excellent mechanical properties, such as SiAlONs [1,2]. Ceramic materials are often amorphous or consist of crystalline grains which are embedded in a glassy matrix, and hence characterization of such materials as well as detection and identification of impurities is not always straightforward. Nuclear magnetic resonance (NMR) spectroscopy has proven to be a powerful analytical technique to analyze ceramic structures, because of its ability to selectively probe the local surrounding of the observed nuclides [3,4,5]. For characterization of a multi-component system, it is crucial to know the exact NMR-interaction parameters of the detected nuclei in the various components, in order to correctly assign and distinguish the NMR signals arising from them. The ‘gold standard’ for the determination of the NMR-interaction parameters, which in the solid state take the form of second-rank tensors [6], is via NMR spectroscopy of single crystals [7].

**Figure 1:**
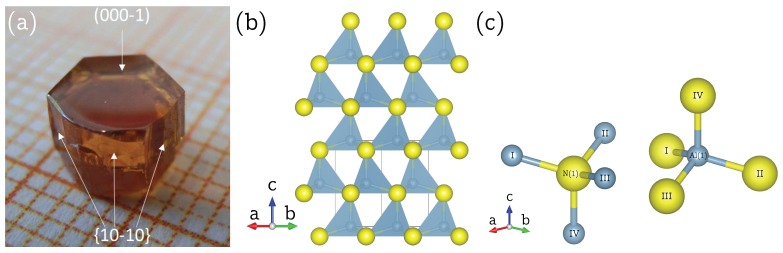
(**a**) Single crystal of aluminum nitride, AlN, with the synthesis described in Reference [8]. The crystallographic *c* axis and the ab plane are indicated by arrows. (**b**) Wurtzite structure of AlN, according to Reference [9], viewed down the crystallographic [11-20] direction. The aluminum atoms (blue-grey) and the nitrogen atoms (yellow), both located at Wyckoff position 2*b*, are tetrahedrally coordinated by each other with one Al-N bond directed parallel to the crystallographic *c* axis. (**c**) Individual, tetrahedrally coordinated, aluminum and nitrogen atom in the crystal structure of AlN, in which the three equal, shorter, bonds Al/N—I/II/III with 1.8891(8) Å and the longer bond Al/N—IV with 1.9029(16) Å along the three-fold rotation axis are highlighted. ${}^{a}$Drawing generated with the V_ESTA_ program [10].

Here, we present the determination of the full chemical shift tensor δ and quadrupole coupling tensor Q for ${}^{27}$Al (I=5/2) and ${}^{14}$N (I=1) in hexagonal aluminum nitride by means of single-crystal NMR spectroscopy. This is made possible by the successful preparation of a macroscopic single crystal of AlN (shown in Figure 1a) using a bulk growth process on a native AlN seed [8]. Aluminum nitride commonly occurs in the hexagonal wurtzite structure, depicted in Figure 1b, with space group $P6_{3}mc$ (No. 186) and two formula units per unit cell [9]. The aluminum as well as the nitrogen atoms are located at Wyckoff position 2b on a three-fold rotation axis parallel to the crystallographic *c* axis. Aluminum is tetrahedrally coordinated by nitrogen and vice versa, with one slightly elongated Al-N bond directed along the three-fold rotation axis, and the other three bonds found in equivalent positions around this axis, as may be seen in Figure 1c. These symmetry constraints have a direct impact on the structure of the NMR tensors, as will be explained in the following.

## 2. Single-Crystal ${}^{14}$N and ${}^{27}$Al NMR

In the solid state, the NMR response of spin I=1/2 is governed by the chemical shift, and by dipolar (direct) couplings between spins [11]. The dipolar couplings between the nuclear spins in the AlN lattice result in homogeneous line broadening and will not be quantitatively evaluated here. Both ${}^{14}$N and ${}^{27}$Al have a spin I>1/2, and therefore, the quadrupolar coupling between the non-symmetric charge distribution of the nucleus and its electronic surroundings also needs to be considered [12]. For a spin *I* in an external magnetic field, 2I NMR transitions exist, which are classified according to their magnetic quantum number *m*. With a particular transition |m〉→|m+1〉 designated by the parameter $k=m+\frac{1}{2}$ [13], the resonance frequency $\nu_{m,m+1}$ of this transition may be described by the following general notation:

(1) $$\nu_{m,m+1}\left(k\right)=\nu_{0}+\nu_{CS}+\nu_{m,m+1}^{\left(1\right)}\left(k\right)+\nu_{m,m+1}^{\left(2\right)}\left(k^{2}\right)$$

For the two transitions of ${}^{14}$N with I=1, the values for *k* are $\pm \frac{1}{2}$. For the five transitions of ${}^{27}$Al with I=5/2, the values for *k* are k=0 for the central transition, and k=±1,2 for the satellite transitions. In Equation (1), $\nu_{0}$ is the Larmor frequency, $\nu_{CS}$ the contribution of the chemical shift (CS), and $\nu_{m,m+1}^{\left(1\right)}\left(k\right)$ and $\nu_{m,m+1}^{\left(2\right)}\left(k\right)$ are the effects of the quadrupolar interaction described by perturbation theory to first and second order, respectively. Magnitude and orientation dependency of the quadrupole interaction may be gauged by the quadrupole coupling tensor Q. Similar to the electrical field gradient (EFG) tensor V, to which it is related by Q=(eQ/ℏ)V, this second-rank tensor is symmetric and traceless, i.e., $Q_{ij}=Q_{ji}$ and $Q_{xx}+Q_{yy}+Q_{zz}=0$. Generally, for NMR spectroscopy of single crystals, it is useful to define three distinct coordinate systems, i.e., the laboratory frame, where the *z* axis is defined by the orientation of the external magnetic field, the crystal lattice (CRY) frame and the principal axis system (PAS). In the wurtzite structure of AlN, nitrogen and aluminum are both situated on a three-fold rotation axis parallel to the crystallographic *c* axis, and therefore the CRY and the PAS frames for ${}^{14}$N and ${}^{27}$Al are identical. In their PAS frame, symmetric tensors take diagonal form. This has the consequence that the tensors cannot change when the two formula units are generated by the symmetry elements of Wyckoff position 2*b*. Therefore, the two ${}^{14}$N and ${}^{27}$Al atoms in the AlN unit cell are practically pairwise magnetically equivalent, even though they do not fulfil the strict equivalence criterion of being connected by either inversion or translation. The Q tensor for both nuclides is hence uniaxial (with asymmetry $\eta_{Q}=\left(Q_{11}-Q_{22}\right)/Q_{33}=0$), and solely defined by the quadrupolar coupling constant $\chi =C_{q}=Q_{33}$:

(2) $$Q^{\mathrm{PAS}}=Q^{\mathrm{CRY}}=\left(\begin{array}{ccc} -\frac{\chi }{2} & 0 & 0\\ 0 & -\frac{\chi }{2} & 0\\ 0 & 0 & \chi \end{array}\right)$$

This tensor is conveniently determined from the separations (’splittings’) of the symmetric doublet k=±0.5 for ${}^{14}$N, and of the satellite transitions (ST’s) with k=±1,2 for ${}^{27}$Al, since these are not affected by the chemical shift and the second-order quadrupolar interaction. Thus, the difference Δν(k) of the resonance frequencies (where we have dropped the m,m+1 subscripts used in Equation (1) for brevity) is:

(3) $$\Delta \nu \left(k\right)=\nu \left(+k\right)-\nu \left(-k\right)=\nu^{\left(1\right)}\left(+k\right)-\nu^{\left(1\right)}\left(-k\right)$$

The contribution of the quadrupolar interaction to first order for η=0 is given by [12]:

(4) $$\nu^{\left(1\right)}\left(k\right)=\frac{3\chi }{2I(2I-1)}\frac{3\cos^{2}\beta -1}{2}k$$

Here, the orientation dependence of $\nu^{\left(1\right)}\left(k\right)$ on the relative orientation of the Q tensor to the external magnetic field is expressed by the Euler angle β, with β being the angle between the eigenvector with the largest eigenvalue, i.e., $Q_{33}=\chi$, and the magnetic field vector.

The contribution of the chemical shift $\nu_{CS}$ to the resonance frequency is gauged by the chemical shift tensor δ. Taking into account the same symmetry arguments as for the Q tensor above, the chemical shift (CS) tensor for ${}^{14}$N and ${}^{27}$Al in AlN is given by:

(5) $$\delta^{PAS}=\delta^{CRY}=\left(\begin{array}{ccc} P & 0 & 0\\ 0 & P & 0\\ 0 & 0 & R \end{array}\right)$$

The weighted trace of δ determines the isotropic chemical shift $\delta_{iso}=1/3\left(\delta_{11}+\delta_{22}+\delta_{33}\right)$ and, similar to the Q tensor, the asymmetry parameter for the CS tensor is $\eta_{CS}=\left(\delta_{22}-\delta_{11}\right)/\Delta \delta =0$. Here, we generally order the tensor components according to the convention $|\delta_{33}-\delta_{iso}\left|\geq \right|\delta_{11}-\delta_{iso}\left|\geq \right|\delta_{22}-\delta_{iso}|$, and make use of the reduced anisotropy $\Delta \delta =\delta_{33}-\delta_{iso}$ [14].

To determine the CS tensor of quadrupolar nuclei with half-integer spins, such as ${}^{27}$Al (I=5/2), it is customary to trace the orientation dependency of the central transition (CT), i.e., the k=0 transition [15]. In cases where the CT signal cannot be resolved [16], the variation of the center of the satellite transitions (and for spin I=1, the center of the doublet with k=±0.5 in all cases) may be traced instead:

(6) $$\nu \left(\Delta k/2\right)=\frac{\nu (+k)+\nu (-k)}{2}=\nu_{0}+\nu_{CS}+\nu^{\left(2\right)}\left(k^{2}\right)$$

For ${}^{14}$N in AlN, the quadrupolar interaction to second order is negligible, and the CS tensor δ may directly be determined from the doublet centers. The CT of ${}^{27}$Al in AlN is, however, affected by the quadrupolar interaction to second order, and this contribution has to be subtracted from the CT line position before δ can be determined. This second-order contribution can be written as [17]:

(7) $$\nu^{\left(2\right)}\left(k^{2}=0\right)=-\frac{1}{64\nu_{0}}{\left[\frac{3\chi }{2I(2I-1)}\right]}^{2}\left[3-4I\left(I+1\right)\right]\left(9\cos^{4}\beta -10\cos^{2}\beta +1\right)$$

After subtracting $\nu^{\left(2\right)}$ from the observed ν, the change of the CT resonance frequency from the Larmor frequency is solely due to the chemical shift. The line position depends on the relative orientation of the magnetic field vector ${\vec{b}}_{0}$ to the tensor $\delta^{\mathrm{CRY}}$ in the crystal frame, which may be compactly expressed by the product [18]:

(8) $$\frac{\nu \left(\Delta k/2\right)-\nu_{0}-\nu^{\left(2\right)}\left(k^{2}\right)}{\nu_{0}}=\frac{\nu_{CS}}{\nu_{0}}\left[\mathrm{ppm}\right]={\vec{b}}_{0}^{T}\cdot \delta^{\mathrm{CRY}}\cdot {\vec{b}}_{0}$$

The determination of the actual quadrupole coupling tensors $Q_{N}$, $Q_{\mathrm{Al}}$ and the chemical shift tensors $\delta_{N}$, $\delta_{Al}$ for ${}^{14}$N and ${}^{27}$Al in aluminum nitride, using the above formalism, is described in the following.

## 3. Results

### 3.1. ${}^{27}$Al Quadrupole Coupling Tensor

**Figure 2:**
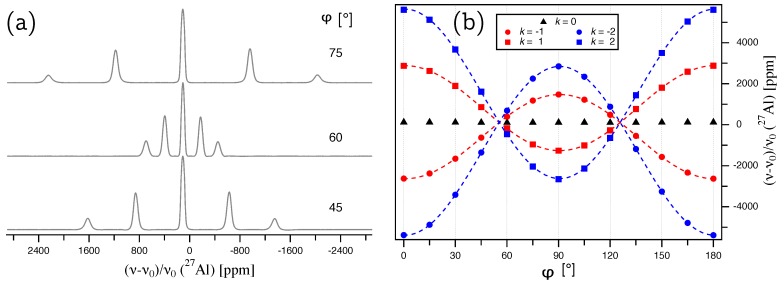
(**a**) ${}^{27}$Al NMR spectra of a single crystal of aluminum nitride, AlN, with the indicated rotation angles φ referring to the full rotation pattern on the right. (**b**) Full rotation pattern over $180^{o}$ for ${}^{27}$Al at Wyckoff position 2*b*, acquired by rotating the AlN crystal counterclockwise by $15^{o}$ around a rotation axis which is perpendicular to the external magnetic field ${\vec{b}}_{0}$, and situated in the crystallographic ab plane of AlN. The zero point of the rotation, φ=0, deviates by $\varphi_{\Delta }=0.65^{\circ }$ from the ideal position where ${\vec{b}}_{0}$ is parallel to the [000-1] direction (see text for details).

A single crystal of aluminum nitride with approximate dimensions of 5×5×4 mm was used for the single-crystal NMR experiments. Since the crystal was grown by a homoepitaxial growth process [8], it is possible to assign the crystal faces to crystallographic planes, as indicated in Figure 1a. It was therefore possible to fix the crystal into in a specific orientation by gluing it with its (10-10) face onto the goniometer axis, which itself is perpendicular to the external magnetic field ${\vec{b}}_{0}$. The crystal was then rotated until the [000-1] direction was parallel to ${\vec{b}}_{0}$. Both orienting procedures involve small misalignments, which can however be quantified by the data analysis, as described below. Representative ${}^{27}$Al NMR spectra are shown in Figure 2a, with the full rotation pattern over $180^{o}$ shown in Figure 2b, which was obtained by rotating the crystal counterclockwise in steps of $15^{\circ }$ using the goniometer gear. The satellite pairs for k=±2, in the following denoted as ST(5/2), and k=±1, in the following denoted as ST(3/2), are symmetrically positioned around the central transition. All ${}^{27}$Al resonance lines are fairly broad, with a full width at half-maximum *fwhm* ≈9 kHz, caused by hetero- and homonuclear dipolar interactions between aluminum and nitrogen atoms in the structure [19].

**Figure 3:**
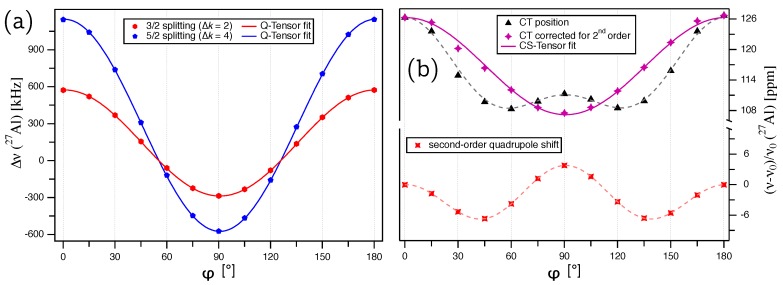
(**a**) Plot of the splittings Δν(k)=ν(+k)−ν(−k) for the ST(3/2) (Δk=2; red) and ST(5/2) (Δk=4; blue) doublets for ${}^{27}$Al in the unit cell of the AlN single crystal. The lines represent the fit of the quadrupole coupling tensor according to Equation (9). (**b**) Plot of the experimentally determined central transition for ${}^{27}$Al (k=0; black), the contribution of the quadrupolar interaction according to Equation (7) (red), and the central transition after subtracting the quadrupolar second-order shift from the experimental data points (purple). The solid purple line represents the fit of the chemical shift tensor according to Equation (11).

The experimentally determined satellite splittings of the ST(5/2) and ST(3/2) doublets in kHz are plotted over the rotation angle φ in Figure 3a. The rotation patterns in both Figure 2b and Figure 3a are mirrored at a position very close to $90^{\circ }$, with the mirror defining the rotation angle for which ${\vec{b}}_{0}$ is situated in the crystallographic ab plane. The deviation $\varphi_{\Delta }$ of the mirror from $90^{\circ }$ quantifies the original misalignment of the [000-1] direction to ${\vec{b}}_{0}$. From the way the crystal is glued on the goniometer axis, we know that the rotation axis must be in the crystallographic ab plane. Also, the above considerations of the effects of crystal symmetry on the tensor structure imply that the eigenvector with the largest eigenvalue ($Q_{33}=\chi$) must point along the three-fold rotation axis, i.e., along the crystallographic *c* axis, which we attempted to align along ${\vec{b}}_{0}$ for the starting point of our rotation pattern. For this situation, the angle β in Equation (4) can be replaced by $\beta \to \varphi -\varphi_{\Delta }$, and the magnitude of the satellite splittings (Equation (3)) an be expressed by:

(9) $$\Delta \nu \left(k\right)=\frac{3\chi \Delta k}{2I(2I-1)}\frac{3\cos^{2}\left(\varphi -\varphi_{\Delta }\right)-1}{2}$$

To determine the quadrupole coupling tensor $Q_{\mathrm{Al}}$ of ${}^{27}$Al, the satellite splittings were simultaneously fitted according to Equation (9) with Δk=2 for the ST(3/2) and Δk=4 for the ST(5/2) splittings. The fit converged on a global solution, giving χ=(1.914±0.001) MHz and $\varphi_{\Delta }={\left(0.65\pm 0.04\right)}^{\circ }$. The quadrupolar coupling constant determined from our single-crystal NMR experiments is in perfect agreement with the previously reported value of χ=1.913 MHz, determined from a static polycrystalline powder sample of AlN [20]. The full $Q_{\mathrm{Al}}$ tensor, with the eigenvalues and corresponding eigenvectors in the PAS frame (Equation (2)), is summarized in Table 1. The quadrupolar asymmetry parameter $\eta_{Q}=0$, and the orientation of the eigenvectors are a consequence of the crystal symmetry, with ${\vec{q}}_{33}$ aligned exactly along the *c* axis and ${\vec{q}}_{11}$, ${\vec{q}}_{22}$ placed in the ab plane.

### 3.2. ${}^{27}$Al Chemical Shift Tensor

To determine the chemical shift tensor $\delta_{Al}$ of ${}^{27}$Al, the contribution of the second-order quadrupolar interaction must be subtracted from the central transition (k=0) line position. In Figure 3b, the ${}^{27}$Al CT is plotted over φ, and the data points clearly show the presence of the quadrupolar-induced shift, which according to Equation (7), contains harmonic terms depending on both $\cos^{4}\left(\beta \right)$ and $\cos^{2}\left(\beta \right)$. Using the results obtained from evaluating the splittings (χ=1.914 MHz and $\varphi_{\Delta }=0.65^{\circ }$), this second-order quadrupole shift can be calculated for each crystal orientation according to Equation (7) with $\beta =\varphi -\varphi_{\Delta }$, see red points in Figure 3b. After subtracting the quadrupole contribution from the experimental points, the remaining variation in CT line position (Figure 3b, purple) is solely caused by the chemical shift tensor, which can be determined from it. Due to the cylindrical symmetry of the tensor and the fact that it does not transform between its PAS and CRY frame (see Equation (5)), the exact orientation of the rotation axis in the crystallographic ab plane of AlN is indeterminate. For simplicity, the rotation axis can be assumed to be parallel to the *b* axis, and the orientation of the magnetic field vector in the CRY frame for each rotation angle φ can be expressed by:

(10) $${\vec{b}}_{0}=\left(\begin{array}{c} \sin(\varphi -\varphi_{\Delta })\\ 0\\ \cos(\varphi -\varphi_{\Delta }) \end{array}\right)$$

Inserting this (and Equation (5)) into Equation (8), we obtain the expression necessary for fitting the data in Figure 3b:

(11) $$\frac{\nu_{CS}}{\nu_{0}}\left[\mathrm{ppm}\right]=\frac{1}{2}\left(P+R\right)+\frac{1}{2}\left(R-P\right)\cos\left[2\left(\varphi -\varphi_{\Delta }\right)\right]$$

For this fit, $\varphi_{\Delta }$ was kept fixed at the value derived from fitting $Q_{\mathrm{Al}}$, and the components of the chemical shift tensor of ${}^{27}$Al determined thereby are P=(107.2±0.3) ppm and R=(126.3±0.3) ppm, with the full tensor listed in Table 1. The isotropic chemical shift of $\delta_{iso}=\left(113.6\pm 0.3\right)$ ppm is in good agreement with a previously reported value [4], which was determined from a polycrystalline sample of AlN under magic-angle spinning (MAS), and after correcting for the second-order quadrupole shift (from the reported line position of 113.3 ppm at a 600 MHz spectrometer [4], the correction of $\nu_{ai}^{\left(2\right)}=-\left(3/500\right)\left(\chi^{2}/\nu_{0}\right)\approx -0.9$ ppm needs to be subtracted), comes out to $\delta_{iso}=114.2$ ppm. The chemical shift asymmetry parameter $\eta_{CS}=0$ and the orientation of the chemical shift eigenvectors follow the same symmetry restrictions as for the quadrupole coupling tensor described above.

### 3.3. ${}^{14}$N Quadrupole Coupling Tensor

**Figure 4:**
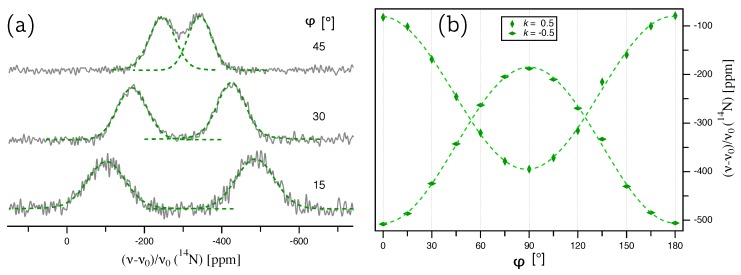
(**a**) ${}^{14}$N NMR spectra of an AlN single crystal, acquired under the same conditions as listed in the caption of Figure 2. The green, dashed lines show the deconvolution of each signal with a Voigt profile, the maxima of which give the line positions plotted on the right. (**b**) Full $180^{o}$ rotation pattern for ${}^{14}$N at Wyckoff position 2*b* in AlN crystal. The point φ=0 deviates by $\varphi_{\Delta }=-0.74^{\circ }$ from the ideal position where ${\vec{b}}_{0}$ is parallel to the [000-1] direction (see text for details).

For the determination of the quadrupole coupling tensor $Q_{N}$ and the chemical shift tensor $\delta_{N}$ of ${}^{14}$N in aluminum nitride, the same AlN crystal (Figure 1a) and goniometer axis as for ${}^{27}$Al was used. Since a change of the solenoid coil was necessary to go from the resonance frequency of ${}^{27}$Al to ${}^{14}$N, the offset angle $\varphi_{\Delta }$ is slightly different and needs to be determined from the data fit again. Representative ${}^{14}$N NMR spectra are depicted in Figure 4a, and at first glance, appear to show much broader lines than the ${}^{27}$Al spectra. In fact, with *fwhm* ≈3 kHz, the resonance lines are only about one third as broad as those of ${}^{27}$Al, since the gyromagnetic ratio of ${}^{14}$N is 3.5 times smaller than that of aluminum, which scales down the homonuclear contribution of the dipolar coupling. The impression of broad lines for ${}^{14}$N is chiefly because the shifts of its k=±0.5 resonances caused by the quadrupolar interaction (≈300 ppm) are much smaller than those of ${}^{27}$Al (≈8000 ppm), since these shifts scale with the quadrupolar moment of the nucleus, which is 20.44 mb for ${}^{14}$N, but 146.6 mb for ${}^{27}$Al [20]. The broad resonance lines of the ${}^{14}$N spectra, combined with the relatively poor signal-to-noise ratio (due to the long relaxation time of $T_{1}=1080$ s [22]) make it difficult to precisely derive the line positions from the spectra. Therefore, all ${}^{14}$N NMR spectra were deconvoluted, assuming combined Lorentz-Gauss functions (so-called Voigt profiles), to reliably obtain the line positions.

**Figure 5:**
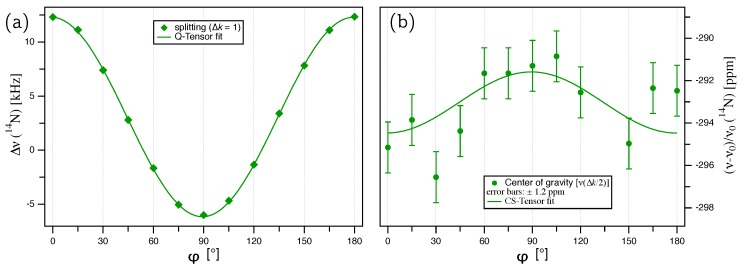
(**a**) Plot of the splittings Δν(k)=ν(+0.5)−ν(−0.5) for ${}^{14}$N from an AlN single crystal. The lines represent the fit of the quadrupole coupling tensor according to Equation (9). (**b**) Plot of the center ν(Δk/2)=[ν(0.5)+ν(−0.5)]/2 for ${}^{14}$N, with the error bars (±1.2 ppm) taken from the Voigt profile fit for each individual signal. The solid green line represents the fit of the chemical shift tensor according to Equation (11).

The splittings of the thus deconvoluted ${}^{14}$N doublets are plotted over the rotation angle φ in Figure 5a. The quadrupole coupling tensor was determined by a fit of these splittings according to Equation (9) with Δk=1, giving the quadrupolar coupling constant χ=(8.19±0.02) kHz and an offset angle of $\varphi_{\Delta }=-{\left(0.74\pm 0.13\right)}^{\circ }$. The full quadrupole coupling tensor, with the eigenvalues and corresponding eigenvectors in the PAS frame (Equation (2)), is summarized in Table 2. The quadrupolar asymmetry parameter $\eta_{Q}=0$, and the orientation of the eigenvectors are identical to the Q tensor of ${}^{27}$Al. So far, only an upper limit of the quadrupolar coupling constant of ${}^{14}$N in AlN was available in the literature, namely χ<10 kHz determined from a polycrystalline powder sample [20].

### 3.4. ${}^{14}$N Chemical Shift Tensor

The chemical shift tensor of ${}^{14}$N can be calculated from the evolution of the center of the doublet with k=±0.5 over the rotation angle, as plotted in Figure 5b. Fitting the data in Figure 5b according to Equation (11), with the offset angle kept fixed at the value derived from the quadrupole coupling tensor fit ($\varphi_{\Delta }=-0.74$ ppm), gives P=−(291.6±0.7) ppm and R=−(294.5±0.6) ppm, with the full tensor listed in Table 2. The data in Figure 5b exhibit quite some scatter; however, it has to be kept in mind that for tracing the anisotropy of the ${}^{14}$N chemical shift in aluminum nitride, we are attempting to extract variations of the order of ≈90 Hz from resonance lines with *fwhm* ≈3 kHz. Despite the scatter, about two thirds of all data points belong to the CS tensor fit function within the error margins of ±1.2 ppm. The resulting isotropic chemical shift $\delta_{iso}=-\left(292.6\pm 0.6\right)$ ppm is in good agreement with the previously reported value of $\delta_{iso}=64.7$ ppm [4], determined from a polycrystalline powder sample under MAS and referenced to an aqueous (NH${}_{4}$)${}_{2}$SO${}_{4}$ solution, with a ’NH${}_{4}^{+}$’ solution resonance shifted −355 ppm relative to the ’NO${}_{3}^{-}$’ solution used here [23]. Similar to the quadrupole coupling tensor, the asymmetry of the CS tensor with $\eta_{CS}=0$, as well as the eigenvector orientation follow the symmetry restrictions of the crystal lattice.

### 3.5. ${}^{14}$N and ${}^{27}$Al DFT Calculations

It has become customary within the solid-state NMR community to augment experimental results by comparing them to predictions derived from calculations using density functional theory (DFT) methods employing periodic plane waves [24]. To check how the quadrupolar coupling constants for ${}^{27}$Al and ${}^{14}$N derived from our precise single-crystal results compare to DFT predictions, we have performed such calculations for aluminum nitride, using the C_ASTEP_ code, see Section 4.3 for computational details. Table 3 shows the quadrupolar coupling constants $\chi_{\mathrm{calc}}$ determined by DFT calculations using the coordinates from X-ray diffraction data reported in the inorganic crystal structure database (I_CSD_) for a selection of different database entries. The variation of these entries concerns mostly the unit cell dimensions (see also below about geometry optimization), which is reflected in the varying unit cell volumes $V_{cell}$ listed in the table. On the left of Table 3, the calculation results are given from directly using the I_CSD_ coordinates, the so-called single-point energy (SPE). We note that for this calculation mode, the DFT algorithm returns $\chi_{\mathrm{calc}}$ values within a wide scatter, mirrored by standard deviations of 37% for ${}^{27}$Al and 73% for ${}^{14}$N. Whereas a single structure might accidentally give numbers for $\chi_{\mathrm{calc}}$ that are practically identical to the experiment, as structure I_CSD_ 34475 does here for AlN, a more systematic exploration would demand to take the arithmetic mean of the eight different structures. These mean values, $\chi_{\mathrm{calc}}{(}^{27}$Al)=3.288 MHz and $\chi_{\mathrm{calc}}{(}^{14}$N)=−26.4 kHz are very far from the experimentally determined values of $\chi {(}^{27}\mathrm{Al})=\left(1.914\pm 0.001\right)$ MHz and $\chi {(}^{14}N)=\left(8.19\pm 0.02\right)$ kHz, with the absolute sign of χ not being available from the experiments.

It is however well documented in the literature that in order to obtain good agreement between DFT and experimental results, a geometry optimization (GO) of the crystal structure is usually necessary [31,32,33]. This was also done for AlN, taking the coordinates of the previously used ICSD database entries as a starting point. It should be noted that for AlN, only the unit cell parameters a,b,c may be geometry optimized, since both aluminum and nitrogen atoms are situated on a crystallographic special position, Wyckoff position 2b. As may be seen from the entries on the right in Table 3, the $\chi_{\mathrm{calc}}$ values are practically independent from the starting point after energy optimization, with a mean of ${\bar{\chi }}_{\mathrm{calc}}{(}^{27}$Al)=1.7913 MHz and ${\bar{\chi }}_{\mathrm{calc}}{(}^{14}$N)=−19.5 kHz. This leads to small standard deviations (0.1% for ${}^{27}$Al and 17% for ${}^{14}$N), which seem to imply a high accuracy of the DFT results. However, the small standard deviations of the GO calculations reflect only on a high precision of the computational algorithm. The accuracy of calculation results is defined by comparison to the experiment [34], and is therefore quite low, since both experimental values (especially that of ${}^{14}$N) are outside the standard deviation of the high-precision ${\bar{\chi }}_{\mathrm{calc}}$ values.

## 4. Materials and Methods

### 4.1. Aluminum Nitride

The single crystal of aluminum nitride shown in Figure 1a was grown at IKZ, using physical vapor transport of bulk AlN in a TaC crucible with radio frequency induction heating. Further details may be found in Reference [8].

### 4.2. Solid-State NMR Spectroscopy

Single-crystal NMR spectra were acquired on a B_RUKER_ Avance-III 400 spectrometer at MPI-FKF Stuttgart, at a Larmor frequency of $\nu_{0}{(}^{27}\mathrm{Al})=104.263$ MHz, and $\nu_{0}{(}^{14}N)=28.905$ MHz, using a goniometer probe with a 6 mm solenoid coil, built by NMR Service GmbH (Erfurt, Germany). The ${}^{27}$Al spectra were recorded with single-pulse acquisition, four scans and a relaxation delay of 20 s. For the ${}^{14}$N spectra a spin–echo sequence [35] was employed to minimize baseline roll and the spectra were recorded with 16 scans and a relaxation delay of 300 s. All spectra were referenced to a dilute Al(NO${}_{3}$)${}_{3}$ solution at 0 ppm. The fit of the rotation pattern and deconvolution of the ^14^N spectra were performed with the program I_GOR_ P_RO_ 7 from WaveMetrics Inc., which delivers excellent non-linear fitting performance.

### 4.3. DFT Calculations

All calculations were run with the C_ASTEP_ density functional theory (DFT) code [36] integrated within the B_IOVIA_ Materials Studio 2017 suite, using the G_IPAW_ algorithm [37]. The computations use the generalized gradient approximation (GCA) and Perdew–Burke–Ernzerhof (PBE) functional [38], with the core-valence interactions described by ultra-soft pseudopotentials [37]. Integrations over the Brillouin zone were done using a Monkhorst–Pack grid [39] of 16×16×8, with a reciprocal spacing of at least 0.025 Å${}^{-1}$. The convergence of the calculated NMR parameters was tested for both the size of a Monkhorst–Pack *k*-grid and a basis set cut-off energy, with the cut-off energy being 1500 eV. Also, the possible contribution of pairwise dispersion interactions was checked by using the Tkatchenko–Scheffler method [40] as implemented in C_ASTEP_, but no improvements were observed. The calculation results reported here therefore do not include dispersion interaction.

Geometry optimization (GO) calculations were performed using the Broyden–Fletcher- Goldfarb–Shanno (B_FGS_) algorithm [41], with the same functional, *k*-grid spacings and cut-off energies as in the single-point energy (SPE) calculations. Convergence tolerance parameters for geometry optimization were as follows: maximum energy 2.0 × 10${}^{-5}$ eV/atom, maximum force 0.001 eV/Å, maximum stress 0.01 GPa/atom, and maximum displacement in a step 0.002 Å. Crystallographic data used in the calculations were taken from literature listed in Table 3.

## 5. Conclusions

In this work, both the chemical shift and quadrupole coupling tensors for ${}^{27}$Al and ${}^{14}$N in aluminum nitride have been determined to high precision by single-crystal NMR spectroscopy. To this end, a homoepitaxially grown AlN single crystal with known morphology was used, which allowed the rotation axis to be determined by optical alignment. Because of the high symmetry of wurtzite-type AlN, one full rotation pattern was sufficient to determine the NMR-interaction tensors in the crystal frame. The three-fold rotation axis on which both atom types are located enforces colinearity of the tensor eigenvectors with the crystallographic coordinate system, which simplifies data analysis. A simultaneous fit for the ST(3/2) and ST(5/2) splittings of ${}^{27}$Al gave the quadrupolar coupling constant $\chi {(}^{27}\mathrm{Al})=\left(1.914\pm 0.001\right)$ MHz, and fitting the ${}^{14}$N doublet splitting resulted in $\chi {(}^{14}N)=\left(8.19\pm 0.02\right)$ kHz. To extract the chemical shift tensor for ${}^{27}$Al, the evolution of the central transition over the crystal rotation was tracked, and the contribution of the second-order quadrupolar shift was subtracted according to the previously determined quadrupolar coupling tensor. A fit over the thus corrected central transition positions resulted in an isotropic chemical shift of $\delta_{iso}=\left(113.6\pm 0.3\right)$ ppm and an reduced anisotropy of $\delta_{\Delta }=\left(12.7\pm 0.6\right)$ ppm. Due to the small quadrupolar moment of ${}^{14}$N, its second-order quadrupolar shift in AlN is negligible, and the chemical shift tensor was directly fitted from the evolution of the ${}^{14}$N doublet centers over the rotation angle. The resulting isotropic chemical shift is $\delta_{iso}=-\left(292.6\pm 0.6\right)$ ppm and the reduced anisotropy is $\delta_{\Delta }=-\left(1.9\pm 1.1\right)$ ppm.

For comparison, the quadrupolar coupling parameters of ${}^{14}$N and ${}^{27}$Al were also calculated using the C_ASTEP_ DFT code for a variety of previously reported X-ray structures. For both calculation strategies, i.e., single-point energy (SPE, where the coordinates are directly taken from XRD), and structures which were geometry optimized (GO) by the DFT code, agreement with the experimental values was relatively poor, leaving room for further improvement of these computational methods.

## Figures and Tables

**Table 1:** Quadrupole coupling tensor $Q_{\mathrm{Al}}$ (left), and chemical shift tensor $\delta_{Al}$ (right) of ${}^{27}$Al in the wurtzite structure of AlN, as determined from single-crystal NMR experiments. The orientation of the corresponding eigenvectors are listed in spherical coordinates (θ,φ) in the hexagonal abc crystal frame CRY. The errors of the experimental values reflect those delivered by the fitting routine.

NMR-Interaction Tensors of ${}^{27}$Al in Aluminum Nitride				
$Q_{11}^{PAS}$	−0.957±0.001 MHz		$\delta_{11}^{PAS}$	107.2±0.3 ppm
$Q_{22}^{PAS}$	−0.957±0.001 MHz		$\delta_{22}^{PAS}$	107.2±0.3 ppm
$Q_{33}^{PAS}=\chi$	1.914±0.001 MHz		$\delta_{33}^{PAS}$	126.3±0.3 ppm
${\vec{q}}_{11}$	90${}^{\circ }$, γ${}^{a}$		${\vec{d}}_{11}$	90${}^{\circ }$, γ${}^{a}$
${\vec{q}}_{22}$	90${}^{\circ }$, γ${}^{a}$ + 90${}^{\circ }$		${\vec{d}}_{22}$	90${}^{\circ }$, γ${}^{a}$ + 90${}^{\circ }$
${\vec{q}}_{33}$	0${}^{\circ }$, 0${}^{\circ }$		${\vec{d}}_{33}$	0${}^{\circ }$, 0${}^{\circ }$
			$\delta_{iso}$	113.6±0.3 ppm
			Δδ	12.7±0.6 ppm
$\eta_{Q}$	0		$\eta_{CS}$	0

${}^{a}$ Indeterminate in the ab plane because of the cylindrical symmetry of the tensor. This situation also makes it impractical to visualize the tensors by plotting the eigenvectors in the unit cell, as has been done before for similar systems [13,16,21].

**Table 2:** Quadrupole coupling tensor $Q_{N}$ (left), and chemical shift tensor $\delta_{N}$ (right) of ${}^{14}$N in the wurtzite structure of AlN, as determined from single-crystal NMR experiments. The orientation of the corresponding eigenvectors are listed in spherical coordinates (θ,φ) in the hexagonal abc crystal frame CRY. The errors of the experimental values reflect those delivered by the fitting routine.

NMR-Interaction Tensors of ${}^{14}$N in Aluminum Nitride				
$Q_{11}^{PAS}$	−4.096±0.009 kHz		$\delta_{11}^{PAS}$	−291.6±0.7 ppm
$Q_{22}^{PAS}$	−4.096±0.009 kHz		$\delta_{22}^{PAS}$	−291.6±0.7 ppm
$Q_{33}^{PAS}=\chi$	8.192±0.020 kHz		$\delta_{33}^{PAS}$	−294.5±0.6 ppm
${\vec{q}}_{11}$	90${}^{\circ }$, γ${}^{a}$		${\vec{d}}_{11}$	90${}^{\circ }$, γ${}^{a}$
${\vec{q}}_{22}$	90${}^{\circ }$, γ${}^{a}$ + 90${}^{\circ }$		${\vec{d}}_{22}$	90${}^{\circ }$, γ${}^{a}$ + 90${}^{\circ }$
${\vec{q}}_{33}$	0${}^{\circ }$, 0${}^{\circ }$		${\vec{d}}_{33}$	0${}^{\circ }$, 0${}^{\circ }$
			$\delta_{iso}$	−292.6±0.6 ppm
			Δδ	−1.9±1.1 ppm
$\eta_{Q}$	0		$\eta_{CS}$	0

${}^{a}$ Indeterminate in the ab plane because of the cylindrical symmetry of the tensor.

**Table 3:** Quadrupolar coupling constant $\chi_{\mathrm{calc}}$ for ${}^{27}$Al and ${}^{14}$N in aluminum nitride, as determined from DFT calculations with the C_ASTEP_ code. Calculations were run using the atomic coordinates of the reported crystal structures directly (single-point energy—SPE), and after geometry optimization (GO) of the unit cell.

I_CSD_	Ref.	$V_{\mathrm{cell}}$	$\chi_{\mathrm{calc}}$ from SPE		$\chi_{\mathrm{calc}}$ from GO	
		[${Å}^{3}$]	${}^{27}$Al [MHz]	${}^{14}$N [kHz]	${}^{27}$Al [MHz]	${}^{14}$N [kHz]
34475	[9]	41.714	1.984	−8.0	1.791	−16.0
34236	[25]	41.724	4.478	43.0	1.791	−22.0
54697	[26]	41.774	4.023	34.0	1.791	−20.0
183638	[27]	41.919	1.836	−12.0	1.795	−16.0
257810	[28]	41.738	3.007	9.0	1.788	−23.0
230434	[29]	41.684	2.101	−12.0	1.788	−24.0
602459	[30]	41.689	4.967	60.0	1.795	−16.0
602460	[30]	41.747	3.910	33.0	1.791	−19.0
$\bar{X}$			3.288	$-26.4^{a}$	1.791	−19.5
σ			1.222 (37%)	19.1 (73%)	0.003 (0.1%)	3.3 (17%)

${}^{a}$ To form the mean, the signs of all individual values were assumed to be negative.
